# Acute Asymptomatic Pericarditis Following Olanzapine Overdose

**DOI:** 10.7759/cureus.97165

**Published:** 2025-11-18

**Authors:** Mohsin Raza, David Ballack, Jasleen Kaur, Shanli Parnia

**Affiliations:** 1 Department of Psychiatry, Dover Behavioral Health System, Dover, USA; 2 Department of Psychiatry and Behavioral Sciences, UF Health St. Johns, University of Florida, St. Augustine, USA; 3 Department of Psychiatry, University of Connecticut Health, Farmington, USA; 4 Department of Internal Medicine, Cimpar, Chicago, USA

**Keywords:** atypical antipsychotic, electrocardiogram, olanzapine, overdose, pericarditis

## Abstract

Olanzapine, a widely utilized atypical antipsychotic, has demonstrated substantial efficacy in managing schizophrenia and bipolar disorder, making it a cornerstone in the treatment of these conditions. Despite its therapeutic benefits, its use has been increasingly associated with adverse cardiovascular outcomes, necessitating a thorough understanding of these risks. Olanzapine is notably associated with metabolic side effects, including significant weight gain, hyperlipidemia, and hyperglycemia, which collectively predispose patients to cardiovascular disease. A rare but important adverse reaction to olanzapine overdose is acute pericarditis. While pericardial effusion with peripheral edema has been described, cases of acute pericarditis as an early manifestation remain exceedingly rare, and the underlying mechanism is poorly understood. We present the case of a male patient in his mid-30s with schizoaffective disorder who intentionally ingested approximately 300 mg of olanzapine in a suicide attempt. On presentation, he was somnolent but hemodynamically stable. Electrocardiography revealed diffuse ST-segment elevations and PR depressions consistent with acute pericarditis, despite the absence of chest pain or dyspnea. Cardiac biomarkers were normal. The patient was treated conservatively with aspirin and colchicine, with subsequent resolution of electrocardiographic changes by hospital day three. Clinicians should be vigilant for signs of acute pericarditis following olanzapine overdose, even in asymptomatic patients, as early electrocardiographic changes may be the only clue. Prompt recognition and management are crucial because untreated pericarditis can progress to complications such as cardiac tamponade or pericardial effusion, which carry a rare but real risk of cardiac arrest. This case highlights an uncommon but clinically relevant cardiovascular complication of olanzapine overdose and underscores the importance of routine cardiac monitoring in such scenarios.

## Introduction

Olanzapine is a widely prescribed second-generation antipsychotic approved by the US Food and Drug Administration (FDA) for the treatment of schizophrenia and bipolar I disorder, including manic or mixed episodes, in adults and adolescents [1]. It is effective for both the positive and negative symptoms of schizophrenia and for mood stabilization in patients with bipolar I disorder [1]. Although the exact mechanism of action is not fully understood, olanzapine is thought to reduce symptoms through modulation of central dopaminergic and serotonergic activity [2]. Compared with typical antipsychotics, olanzapine is associated with a lower incidence of extrapyramidal symptoms, including tardive dyskinesia [3,4]. Its most commonly reported side effects include sedation, weight gain, somnolence, lightheadedness, and dizziness [3,4].

There have been a few case reports describing the development of peripheral edema and pericardial effusion with routine olanzapine use, but earlier clinical manifestations, particularly in the context of acute overdose, have not been well documented [5]. Serious cardiovascular events with olanzapine treatment are infrequent [6]. Nevertheless, QTc prolongation is common among antipsychotics and varies in severity [7]. Orthostatic hypotension is the most frequently observed vascular adverse event, occurring in 1-5% of patients receiving oral or intramuscular olanzapine compared with 0.2% of those receiving placebo in clinical trials [8].

Here, we describe the case of a male patient in his mid-30s who developed asymptomatic acute pericarditis, detected by electrocardiogram (EKG), following intentional olanzapine overdose. Acute pericarditis is defined as sudden inflammation of the pericardium sac that surrounds the heart. Treatment typically targets the underlying etiology and focuses on pain relief and resolution of inflammation [9-11]. To our knowledge, this represents the first reported case of acute pericarditis as an early cardiovascular complication of olanzapine overdose.

## Case presentation

Mr. W is a male in his mid-30s with a significant psychiatric history of schizoaffective disorder, depressive type, and post-traumatic stress disorder (PTSD). His past medical history is notable for diabetes mellitus, managed with metformin, and hyperlipidemia, managed with atorvastatin. He presented to the hospital following an intentional suicide attempt and was subsequently placed under an involuntary psychiatric hold for further evaluation. He reportedly ingested approximately 30 tablets of 10 mg olanzapine in a single sitting. Olanzapine had been prescribed as a once-daily medication by a psychiatrist in 2017, and based on his subjective history, he was likely noncompliant.

**Figure 1:**
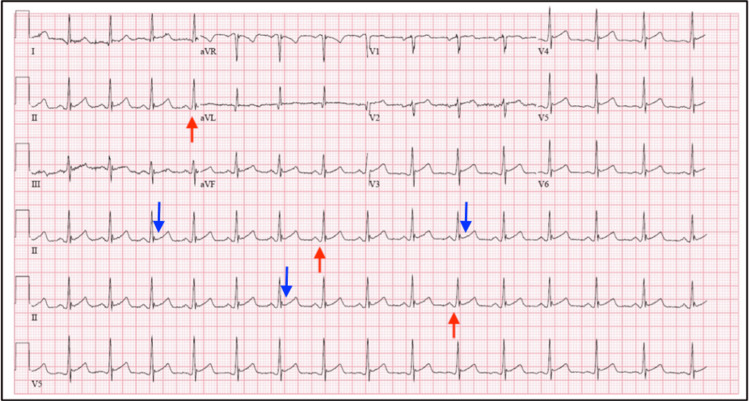
Electrocardiogram (EKG). (1) The blue arrow indicates diffuse ST-segment elevation. (2) The red arrow indicates PR-segment depression.

On presentation to the emergency department (ED), Mr. W was somnolent and difficult to arouse but had normal vital signs. Initial laboratory testing, including a complete blood count (CBC), thyroid-stimulating hormone (TSH), and comprehensive metabolic panel (CMP), was largely unremarkable; however, the lipid panel results were elevated (Table 1). Urine toxicology and blood alcohol levels were negative. Electrocardiography (EKG) done approximately six hours after overdose demonstrated diffuse ST-segment elevations and PR-segment depressions in all leads, consistent with acute pericarditis, with normal troponin levels (Figure 1). Other common causes of ST-segment elevation, such as early repolarization, left ventricular hypertrophy, and benign ST-segment elevation, were considered, but the EKG presentation was classic for acute pericarditis as stated above. Computed tomography of the head performed due to altered mental status revealed no acute or chronic abnormalities. Due to the patient’s somnolent state, we were unable to determine whether he had symptoms of chest pain or dyspnea. He received one dose of naloxone and 1 L of normal saline in the ED. His vital signs remained stable throughout his ED stay. According to collateral history, the patient had no recent viral prodrome, and there was no history of prior cardiac disease or chest trauma. A prior EKG from a psychiatric hospitalization two weeks earlier did not show any ST-segment changes. Chart review revealed he had undergone two electroconvulsive therapy (ECT) sessions for depression during that admission and was discharged on aripiprazole 10 mg PO daily. 

**Table 1:** Laboratory values. ALP: Alkaline Phosphatase; ALT: Alanine Aminotransferase; AST: Aspartate Aminotransferase; BUN: Blood Urea Nitrogen; CBC: Complete Blood Count; CMP: Comprehensive Metabolic Panel; eGFR: Estimated Glomerular Filtration Rate; HDL: High-Density Lipoprotein; LDL: Low-Density Lipoprotein; MCV: Mean Corpuscular Volume; MCH: Mean Corpuscular Hemoglobin; MCHC: Mean Corpuscular Hemoglobin Concentration; MPV: Mean Platelet Volume; RBC: Red Blood Cell Count; RDW: Red Cell Distribution Width; TSH: Thyroid-Stimulating Hormone; WBC: White Blood Cell Count

Test Name Result		Units	Flag	Reference Range
CMP				
Total Globulin	2.2	g/dL		1.9-3.7
Sodium	141	mmol/L		137.0-145.0
Potassium	4.7	mmol/L		3.5-5.1
Chloride	105	mmol/L		98.0-107.0
CO_2_	28	mmol/L		22.0-29.0
Anion Gap	8	mEq/L		8.0-16.0
Glucose	122	mg/dL	High	70.0-100.0
Calcium	9.8	mg/dL		8.4-10.2
AST	39	U/L		0.0-40.0
ALT	37	U/L		0.0-40.0
ALP	107	U/L		38-126
Albumin	4.7	g/dL		3.5-5.0
Total Bilirubin	0.7	mg/dL		0.20-1.30
BUN	18	mg/dL	High	7.0-17.0
Creatinine	1	mg/dL		0.52-1.04
BUN/Creatinine Ratio	21	-		6.0-22.0
Total Protein	6.9	g/dL		6.3-8.2
eGFR	84	mL/min/1.73m2		
Albumin/Globulin Ratio	2.1	ratio		1.0-2.5
Lipid Panel				
HDL Cholesterol	49	mg/dL		35.0-80.0
Total Cholesterol	256	mg/dL	High	<200.0
Triglycerides	210	mg/dL	High	0.0-150.0
Cholesterol/HDL Ratio	6.4			
LDL Cholesterol	131	mg/dL	High	0.0-100.0
Thyroid Function				
TSH	1.1	uIU/mL		0.3-4.2
CBC with Automated Differential				
WBC	11.2	k/uL	High	4.0-11.0
RBC	4.79	M/Ul		4.3-5.7
Hemoglobin	15.7	g/dL		14.0-18.0
Hematocrit	47.2	%		42.0-50.0
MCH	32.8	pg		25.1-33.3
MCHC	33.3	g/dL		32.5-34.5
Platelet Count	352	k/uL		165.0-429.0
Neutrophils (%)	61.1	%		40.0-70.0
Monocytes (%)	5	%		0.0-12.0
Lymphocytes (%)	32.3	%		20.0-45.0
Eosinophils (%)	1.4	%		0.0-7.0
Basophils (%)	0.3	%		0.0-1.0
Neutrophils (Absolute)	6.83	× 10³/µL	High	1.9-5.9
Monocytes (Absolute)	0.56	× 10³/µL		0.2-0.7
Eosinophils (Absolute)	0.15	× 10³/µL		0.1-0.5
Basophils (Absolute)	0.03	× 10³/µL		0.0-1.1
MPV	7.0	fL		7.0-9.0
MCV	99	fL	High	76.0-98.0
RDW	15.3	%	High	10.9-14.6

Mr. W was admitted to the medical unit with a primary diagnosis of acute metabolic encephalopathy secondary to drug overdose. Upon admission, he was confused and somnolent, but there were no structural abnormalities noted on brain imaging. He received intravenous fluids (Ringer’s lactate). By the second day of hospitalization, as his mental status improved, Mr. W reported a two-year history of recurrent depressive episodes and multiple unsuccessful antidepressant trials. Following a recent discharge from another hospital, he had been prescribed aripiprazole 10 mg PO daily and benztropine 1 mg PO daily, with instructions to discontinue olanzapine. He had not sought psychiatric care or been hospitalized for mental health issues in the preceding two years. Figure 1 presents the patient's EKG.

The patient reported no use of psychotropic or medical prescriptions apart from olanzapine and benztropine. He acknowledged nonadherence to aripiprazole, which had been prescribed during his most recent psychiatric hospitalization. His antidepressant therapy had been discontinued approximately two weeks prior to admission. His medical history was otherwise notable for type 2 diabetes mellitus and hyperlipidemia, for which he was prescribed metformin and atorvastatin, though he admitted to irregular adherence. He denied any intentional overdose involving these medications and had no other systemic illnesses.

**Figure 2:**
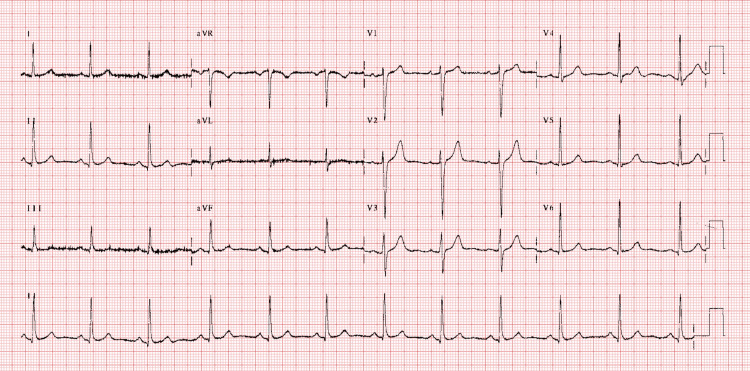
Repeat EKG.

Mr. W denied chest pain, dyspnea, headache, or orthopnea. He was started on aspirin 81 mg PO daily and colchicine 0.5 mg PO twice daily by the primary medical team. A repeat EKG (Figure 2) on hospital day three demonstrated resolution of ST-segment and PR-segment changes. He was subsequently transferred to the psychiatric unit for further behavioral assessment and management.

## Discussion

This case highlights acute asymptomatic pericarditis as a complication most likely related to an intentional olanzapine overdose of approximately 300 mg. Other contributing factors that may have exacerbated the patient’s pericarditis include hypercholesterolemia and obesity [3].

Atypical antipsychotics have largely replaced typical (first-generation) antipsychotics in clinical practice because of their improved safety profile; however, their cardiac safety remains a significant concern [4]. Pericarditis has been previously reported in association with clozapine [4,5]. Murko et al. described a case of pericardial effusion and pericarditis in a male patient with chronic paranoid schizophrenia who had been treated with clozapine for seven years [5]. Olanzapine is structurally similar to clozapine, a thienobenzodiazepine widely used in the treatment of schizophrenia and bipolar disorder [7]. Daly et al. reported a case of pericardial fluid re-accumulation in a patient who continued clozapine therapy despite initial effusion [12]. Rarely, olanzapine has also been associated with the development of dilated cardiomyopathy [13].

Minns et al. noted that the most common cardiovascular effects following atypical antipsychotic overdose include tachycardia and QTc prolongation [14]. There are no specific antidotes for these overdoses, unlike agents such as flumazenil for benzodiazepines or acetylcysteine for acetaminophen. Most cases are managed supportively, including with nonsteroidal anti-inflammatory drugs (NSAIDs) and colchicine [14,15]. In this case, the pericarditis was detected in the context of an overdose, and it remains unknown if this reaction can occur at therapeutic doses. Clozapine, the only antipsychotic with an indication for treatment-resistant schizophrenia, carries a boxed warning for increased risk of fatal myocarditis [16]. Myocarditis refers to inflammation of the heart muscle, whereas pericarditis involves inflammation of the pericardial lining. In both conditions, the immune system mounts an inflammatory response, either to neutralize infection or in response to another trigger; in this case, likely a direct effect of antipsychotic overdose [16].

The mechanisms underlying olanzapine-induced pericarditis remain unclear and have not been fully described in the literature. Some reports describe peripheral edema secondary to olanzapine-induced pericardial effusion, possibly due to its receptor profile or an allergic mechanism; however, the distinction between these processes has not been fully elucidated [2]. Comparison with clozapine is reasonable given their structural and pharmacologic similarities. Clozapine has been more frequently associated with pericarditis, pericardial effusion, and polyserositis [12,15]. Autopsy findings from clozapine-treated patients who developed fatal myocarditis have revealed eosinophilic infiltrates with myocytolysis, consistent with an acute drug reaction [16]. The most widely accepted explanation is an IgE-mediated (type I) hypersensitivity response [13].

This case has several limitations. The patient did not undergo evaluation for eosinophilia, serum IgE levels, or pericardial fluid analysis, as no effusion was detected and only inflammatory changes were observed on ECG. Nonetheless, pericardial inflammation can precede effusion, and the clinical course was consistent with early pericardial involvement. Furthermore, the absence of echocardiographic assessment limits the evaluation of cardiac function and the detection of subtle pericardial effusion.

To date, and to the best of our knowledge, only two published cases have reported olanzapine-associated pericardial effusion. Alagha et al. described a symptomatic patient who developed simultaneous pericardial and pleural effusions three weeks after starting olanzapine; pleural fluid analysis revealed an exudative effusion with predominantly eosinophils and peripheral eosinophilia, suggesting an allergic mechanism [17]. The second case involved a patient with mania treated with olanzapine after discontinuing duloxetine and trazodone; within two days, the patient developed peripheral edema of all extremities, and a two-dimensional echocardiogram demonstrated a pericardial effusion [7]. Laboratory findings were normal without eosinophilia, suggesting a distinct mechanism, similar to our case.

## Conclusions

Olanzapine is a commonly used antipsychotic for a variety of psychiatric disorders. Although cardiovascular adverse effects are uncommon, they can be serious and potentially life-threatening. Physicians should evaluate patients carefully before initiating olanzapine and weigh the risks versus benefits, particularly in individuals at increased risk for suicide or with pre-existing metabolic or cardiovascular conditions.

The mechanism by which olanzapine overdose induces acute asymptomatic pericarditis remains unclear. This case highlights the importance of maintaining a high index of suspicion for pericarditis in patients presenting with olanzapine toxicity, even when typical symptoms are absent. Early cardiac monitoring, prompt recognition, EKG, and appropriate management are critical to prevent serious complications. Further clinical reports and research are needed to clarify the underlying mechanisms and to guide standardized monitoring and treatment protocols. Given the rarity of this adverse effect, clinicians are encouraged to report similar cases to regulatory systems such as the US FDA MedWatch program to improve understanding and surveillance of olanzapine-related cardiovascular complications.
